# Insight into the Advances in Clinical Trials of SARS-CoV-2 Vaccines

**DOI:** 10.1155/2022/6913772

**Published:** 2022-02-09

**Authors:** Syed Mohammed Basheeruddin Asdaq, Shahamah Jomah, Syed Imam Rabbani, Ali Musharraf Alamri, Salman Khalaf Salem Alshammari, Badr Sami Duwaidi, Majed Sadun Alshammari, Abdulhakeem S. Alamri, Walaa F. Alsanie, Majid Alhomrani, Nagaraja Sreeharsha, Mohd. Imran

**Affiliations:** ^1^Department of Pharmacy Practice, College of Pharmacy, AlMaarefa University, Dariyah, Riyadh 13713, Saudi Arabia; ^2^Pharmacy Department, Dr. Sulaiman Al-Habib Medical Group, Riyadh, Saudi Arabia; ^3^Department of Pharmacology and Toxicology, College of Pharmacy, Qassim University, Buraydah 51452, Saudi Arabia; ^4^King Abdulaziz Medical City, Ministry of National Guard, Riyadh, Saudi Arabia; ^5^Department of Clinical Laboratory Sciences, The Faculty of Applied Medical Sciences, Taif University, Taif, Saudi Arabia; ^6^Centre of Biomedical Sciences Research (CBSR), Deanship of Scientific Research, Taif University, Taif, Saudi Arabia; ^7^Department of Pharmaceutical Sciences, College of Clinical Pharmacy, King Faisal University, Al-Ahsa, Al Hofuf 31982, Saudi Arabia; ^8^Department of Pharmaceutics, Vidya Siri College of Pharmacy, Off Sarjapura Road, Bengaluru 560035, Karnataka, India; ^9^Department of Pharmaceutical Chemistry, Faculty of Pharmacy, Northern Border University, P.O. Box 840, Rafha 91911, Saudi Arabia

## Abstract

Severe acute respiratory syndrome coronavirus-2 (SARS-CoV-2) has thrown a challenge to the scientific community. Several interventions to stop or limit the spread of infection have failed, and every time the virus emerges, it becomes more contagious and more deadly. Vaccinating a significant proportion of the population is one of the established methods to achieve herd immunity. More than 100 COVID-19 vaccines have been designed and tested against the virus. The development of a new vaccine takes years of testing, but due to the pandemic, healthcare authorities have given emergency use authorization for a few vaccines. Among them are BioNTech and Moderna vaccines (mRNA based); ChAdOx1, Gam-COVID-Vac, Janssen vaccines (vector-based); CoronaVac, COVAXIN (virus inactivated); and EpiVacCorona vaccine (viral peptide). Mixtures of vaccines are also being tested to evaluate their efficacy against mutant strains of SARS-CoV-2. All these vaccines in clinical trials have shown robust production of neutralizing antibodies sufficient to prevent infection. Some of the vaccinated people reported serious complications. However, no definitive relationship could be established between vaccination administration and the occurrence of these complications. None of the COVID-19 vaccines approved to date have been found to be effective against all of the SARS-CoV-2 variants.

## 1. Introduction

Human coronaviruses are respiratory viruses that were discovered in the 1960s, and seven strains have been identified to date [1]. Some human coronaviruses, like HCoV-229E, HCoV-OC43, HCoV-NL63, and HCoV-HKU1, are known to cause mild respiratory illnesses [2]. However, more infectious and dangerous strains such as severe acute respiratory syndrome coronavirus (SARS-CoV) and Middle East Respiratory Syndrome Coronavirus (MERS-CoV) emerged in 2002 and 2012, respectively [3]. The severe acute respiratory syndrome coronavirus-2 (SARS-CoV-2) was discovered in December 2019 and is the cause of coronavirus disease 2019 (COVID-19). It is related to SARS-CoV and MERS-CoV in terms of phylogeny [1, 3].

The first case of COVID-19 was reported from Wuhan, China, in 2019. It soon spread to other parts of the world and was ultimately declared a pandemic in March 2020 by the World Health Organization (WHO). It has impacted hundreds of millions of people and claimed the lives of nearly three million [4]. It is a highly contagious disease, transmitted through respiratory droplets and direct contact with infected people. Also, the virus can cause a wide range of illnesses, from self-limited mild respiratory illness (80% of cases) to severe respiratory failure, multiple-organ failure, and death [5]. Elderly people, pregnant women, and people with underlying medical conditions are at higher risk of developing severe complications from COVID-19 [6].

With the urgent need to control the COVID-19 pandemic, the Food and Drug Administration (FDA) has created the Coronavirus Treatment Acceleration Program (CTAP), aiming to move new therapies as soon as possible to patients. A total of 490 trials have been reviewed by the FDA, and among them, 9 drugs have received emergency use authorization, and only one (Remdesivir) has been approved by the FDA for the treatment of COVID-19 in adults and paediatric patients aged more than 12 [7]. However, their efficacy was inconsistent in different study settings. In the absence of effective and safe therapeutic interventions for COVID-19, preventing the development of severe illness was considered one of the most suitable options [8].

Many studies and trials on vaccine development started immediately after the identification of the full genomic sequence of SARS-CoV-2. The studies to design a vaccine began in the early 2020s and are now progressing at a lightning pace. If, in recent times, a safe and effective COVID-19 vaccine is developed, then this could create history in modern medicine [9]. Several technological approaches have been adopted for developing the COVID-19 vaccines, and the most important ones are attenuated, protein, vector, and mRNA-based. Many vaccines have received emergency use authorization to administer the jab to the most vulnerable groups of the population. The Pfizer-BioNTech COVID-19 vaccine was the first COVID-19 vaccine to be authorized for emergency use by the FDA in December 2020. Subsequently, Moderna COVID-19 Vaccine and Janssen COVID-19 Vaccine have been authorized for emergency use by the FDA for the prevention of COVID-19. Moreover, other vaccines are in the authorization process [10].

With the aim of achieving herd immunity, countries have started a mass inoculation program with different types of COVID-19 vaccines. The safety and efficacy of vaccines depend to a large extent on the design and the process of development, as well as on the individual response shown by the host system. Hence, it is extremely essential to know all the effects that were observed during vaccine testing [9, 10]. Several studies in the past have highlighted the important safety and efficacy parameters observed in this population in a clinical setting. Some vaccine trial data suggested increased chances of abortion and hemorrhagic complications in certain groups of participants [11]. This crucial information is essential for establishing the complete efficacy and safety of the vaccine but was not elaborately discussed in the previous studies [12]. Therefore, the present study was planned with the purpose of compiling the critically important scientific data published by research organizations during the conduct of clinical trials and then analyzing it in a way that will help medical professionals and the public decide the most suitable vaccine for preventing COVID-19.

## 2. Methods

An online review of literature was conducted on PubMed, Google Scholar, and Science Direct websites using keywords such as “COVID-19,” “Vaccine,” “Clinical Data,” “Trials,” “Adverse Reaction,“ and “Mechanism.“ The review included clinical trials conducted from the beginning of 2020, coinciding with the reports of the successful design of the COVID-19 vaccine, until the end of July 2021 [9]. The review resulted in more than 3000 total articles. However, only 45 articles were selected for the present study based on the inclusion criteria. The authors independently reviewed the titles, abstracts, and text of the articles. The information such as English language, study center, number of subjects, study design, study protocol, dose, duration, route of administration, ethical approval, statistical methods, and biochemical estimations were considered the critical parameters for evaluating the content and were considered the inclusion criteria. Only those articles containing this information were selected for the analysis. Articles having this information were only included for further analysis [8].

## 3. Data Analysis

**Figure 1:**
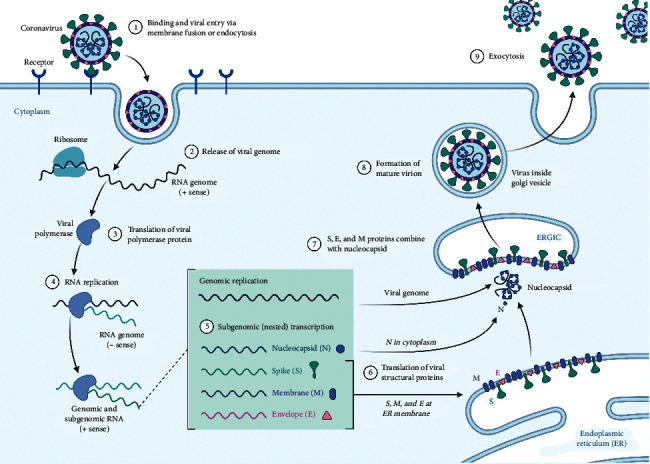
Life cycle of SARS-CoV-2. Reprinted from “Coronavirus replication cycle” by BioRender.com (2020). Retrieved from http://app.biorender.com/biorender-templates.

SARS-CoV-2 is an RNA virus that targets the angiotensin converting enzyme-2 of host cells, and this action occurs through specialized binding glycoproteins called spike proteins. This interaction is crucial for the progression of infection. The transmembrane serine protease of the host cell facilitates the entry of viruses. Inside the host cell, the RNA of the virus modulates the function of synthesizing different components, such as viral polyproteins, nucleic acids, and structural proteins [13]. Finally, these components are assembled and released to attack a new host cell. The steps involved in the life cycle of SARS-CoV-2 are considered vital targets to limit pathogenesis (Figure 1). Almost all the vaccines designed and developed for COVID-19 are aimed at spiking proteins. Extensive research and testing for these vaccines began in early 2020 [14].

**Figure 2:**
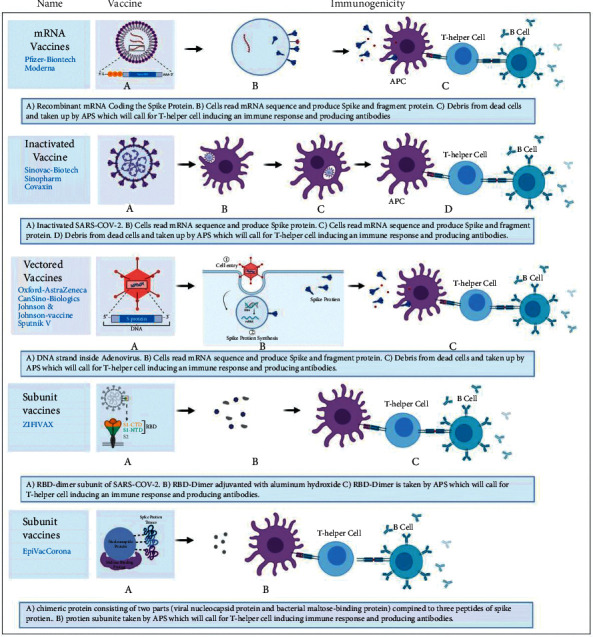
COVID-19 vaccines and their mechanism of activating immune system. Created with BioRender.com.

There are more than 100 COVID-19 vaccines under various stages of development and clinical evaluation. These vaccines can be classified as protein subunits, inactivated viruses, DNA-based, RNA-based, viral vectors, and live-attenuated vaccines (Figure 2). The United States, China, the European Union, the United Kingdom, and India are the top five countries that have done mass inoculation so far. Countries are using different types of vaccines to inoculate their population. Currently, no vaccine is certified to be superior/inferior in terms of safety and efficacy [14, 15].

mRNA-based vaccines can be classified into two forms: nonreplicating mRNA and self-amplifying mRNA vaccines. The mRNAs are designed and synthesized in the laboratory. They are incorporated into liposomes, so that the mRNA can be carried into the cell and prevented from degradation. Once inside the cell, the mRNA is translated into ribosomes to produce specific proteins (spike glycoproteins). The spike proteins are recognized by immune cells and stimulate antibody production [16].

DNA vaccines are also referred to as nucleic acid/genetic vaccines. These vaccines contain the plasmid DND, derived from eukaryotes. After entering cells, the DNA is transcribed and translated to produce specific proteins. This stimulates the immunological system of the host to produce both specific and nonspecific responses, leading to the generation of antibodies [17]. The attenuated and viral component containing vaccines are designed in such a way that they trigger the immune cells to produce neutralizing antibodies [18]. The following sections summarize the important COVID-19 vaccines with a brief description of their characteristics. The analysis of the clinical trial data is represented in Tables 1–12.

### 3.1. BioNTech COVID-19 Vaccine

BioNTech has two vaccine candidates, such as BNT162b1 and BNT162b2 (Table 1). These vaccines are based on mRNA technology and are derived with the modification of nucleosides and formulated in lipid. The mRNA codes for the receptor-binding domain of spike proteins. According to reports available, the serum IgG antibody concentration after the first dose was found to be comparable to the level observed in COVID-19 recovered patients [19]. Further, a dose-dependent response in the level of IgG antibodies was measured when 10 *μ*g and 30 *μ*g of the vaccine were tested in the study population. The elevation in the level of neutralizing antibodies was found to be 10X and 45X, respectively, for the two doses when compared to serum levels of COVID-19 patients. However, a further increase in the dose (100 *μ*g) did not show any additional rise in serum IgG concentration [20].

The administration of BNT162b1 induced functional CD4^+^ and CD8^+^ in 95.2% of human volunteers. The CD4^+^ cells were found to target specifically the SARS-CoV-2 RBD [21]. A similar type of response (94.6%) was also observed when BNT162b2 was administered to study participants older than 16 years. After second dose administration, the immunity response showed a boost, especially in young and older adults, but in people between 65 and 85 years old, the immunological response was found to be weak. Many of the study members indicated manageable common adverse reactions, including a grade 3 decrease in lymphocyte count and grade 2 neutropenia [22]. A few serious adverse events such as atherosclerosis, cardiac arrest, and paroxysmal ventricular arrhythmia resulting in death were reported. However, cardiovascular and thrombotic events were also observed in placebos due to unknown causes [23].

### 3.2. CoronaVac COVID-19 Vaccine

This vaccine was developed by a Chinese pharmaceutical firm called Sinovac Life Sciences. The inactivated strains of SARS-CoV-2 were created and purified from Vero cell lines and are used in the vaccine production [24]. Two doses of the vaccine (3 *μ*g and 6 *μ*g) were tested. The lower dose (3 *μ*g) produced 88% of seroconversion rate, while the higher dose (6 *μ*g) indicated 100% seroconversion rate. The two-dose vaccine needs to be administered at an interval of 14 days [25]. On 28th day of vaccination, both the doses (3 and 6 *μ*g) stimulated the production of neutralizing antibodies but the higher dose (6 *μ*g) of vaccine showed better immunogenic response [26]. The vaccine administration did not show any serious adverse reaction except in one case, where, within 48 hours of first shot, a volunteer experienced hypersensitive reaction such as urticaria [27]. The phase III analysis suggested that the vaccine administration produced 50% protective efficacy in preventing symptomatic infection, 78% in preventing mild cases requiring treatment and 100% in preventing severe form of infection (Table 2) [28].

### 3.3. ChAdOx1 nCoV-19 Vaccine

This is a vector-based vaccine, designed and developed by Oxford University (Table 3). The genetic sequence for the full-length structural glycoprotein of SARS-CoV-2 with tissue plasminogen is incorporated into a nonreplicating simian adenovirus vector called ChadOx1. After administration, the codons express the genes for the synthesis of spike protein by host cells. These glycoproteins have antigenic properties and stimulate the production of antibodies. The first dose of the vaccine required 28 days to show peak antibody levels in the serum and was found to remain for 56 days [47].

The data from the clinical studies suggested that the vaccine is better tolerated by older adults. The second dose produced a better serological response in terms of elevated antibody levels and was found to be independent of participants' age. Vaccine efficacy was found to be high in volunteers receiving a low dose initially followed by a standard second dose. The vaccine in the study participants produced nonserious adverse reactions. A few cases of hemolytic anemia and transverse myelitis were reported in vaccinated people, and the independent expert committee ruled out any direct relationship with the vaccine [17, 29].

Thromboembolic events observed in AZD1222 vaccinated individuals have been extensively studied. The reports, after analyzing all the data, suggested that, in most of the patients who showed this adverse event, the presence of anti-platelet factor4-heparin antibodies was identified. The event has occurred due to the formation of an adenovirus-platelet-leukocyte complex in patients receiving the AZD1222 vaccine [30].

### 3.4. Moderna mRNA Vaccine

This pharmaceutical company's COVID-19 vaccine is also based on mRNA technology (Table 4). The mRNA was designed to encode for S-2P antigens, which are SARS-CoV-2 glycoproteins having a transmembrane and an S1–S2 cleavage site. After vaccination, the host immune system was found to identify the antigens and produce IgG antibodies with a seroconversion rate of 100% by day 15 [48]. A dose-dependent enhancement in the IgG antibodies was observed in the study participants. Three doses of the vaccine (25, 50, and 100 *μ*g) were tested. In the phase-I clinical trials, 25 *μ*g and 50 *μ*g were tested, while, in phase-III, a higher dose (100 *μ*g) was administered. Both the combinations of doses, such as 25 *μ*g + 50 *μ*g and 25 *μ*g + 100 *μ*g, produced a dose-dependent increase in the CD4+ involving Th1 helper T cells. The phase III studies indicated that the level of protection against COVID-19 was 94.1%. The vaccine showed consistency in the protective action irrespective of the age (18–65 years and ≥65 years), sex, and ethnicity of participating members. The adverse reactions recorded for the different doses of vaccine were found to be the same as those observed with placebo and after any vaccination [31].

### 3.5. BBIBP-CorV Vaccine

It is an inactivated virus vaccine developed by Beijing Institute of Biological Products (Table 5). The strains of 19nCoV-CDC-Tan-HBO_2_ were inactivated and purified by passing through Vero cell lines. Mass production of the vaccine was done in basket reactor, and a novel carrier was used to deliver the genetic sequence in the host cells. Three doses of the vaccine, namely, 2, 4, and 8 *μ*g, were tested [49]. All the doses of vaccine produced higher seroconversion on day 28, while medium dose (4 *μ*g) produced this effect on day 21 and highest dose (8 *μ*g) on day 14. Further, not-much variation was observed in the levels of antibodies between medium (4 *μ*g) and highest (8 *μ*g) tested dose of vaccine on day 28. The serological analysis also indicated the higher concentrations of neutralizing antibodies mostly in younger adults compared to older adults. All the participants involved in vaccine testing reported mild side effects such as fever (>38.5 C) [32].

### 3.6. BBV152 Vaccine

The vaccine was developed by an Indian company (Bharath Biotech) (Table 6). The vaccine contains a whole virion-*β*-propiolactone-inactivated SARS-CoV-2. The strains of the virus are formulated in Algel molecules that assist in preventing the vaccine's degradation and entry into the host cells. Two doses of the vaccine, such as 3 *μ*g and 6 *μg*, were tested. The dosage regimen was followed with a gap of 14 days between two doses. The neutralizing antibodies were found to have peaked on day 56 [33]. Antibodies against spike (S1) proteins, RBD and nucleocapsid proteins of SARS-CoV-2 such as CD4+, CD27+, Th1, and Th2 dependent antibody isotopes were present in the study participants. The seroconversion rates of neutralizing antibodies for 3 *μ*g and 6 *μ*g were found to be 92.9% and 98.3%, respectively. All the members of the study reported mild, tolerable side effects (Grade 2/3) and none experienced any serious complications [34].

### 3.7. RBD-Based Protein Subunit Vaccine

A Chinese biotechnological firm has designed and developed the vaccine, especially against the variants of SARS-CoV-2 (Table 7). The spike proteins' RBD dimer was used as the target after carefully analyzing the sequence of the mutated strains of the virus. The vaccine needs to be administered in three doses. The serum analysis indicated the level of neutralizing antibodies increased by 1.6–2.8-fold [35]. However, we are still awaiting complete data on the efficacy of the vaccine against the variants of SARS-CoV-2. Also, studies to confirm the type of neutralizing antibodies and their extent of seroconversion rate are in progress. The vaccine tested in different phases of clinical trials did not show major adverse reactions in the study participants [36].

### 3.8. EpiVacCorona Vaccine

This vaccine is developed by a Russian Biological Research Center (Vector Institute) (Table 8). A synthetic viral peptide was prepared that resembles the SARS-CoV-2 component. The administration of this component is reported to trigger the antigenic response in the body, stimulating the production of antibodies [37]. The vaccine is being tested on the population over 18 years of age. The data from clinical trials indicated that the administration of two doses of vaccine activated the production of antibodies. We are still awaiting more details about the efficacy and safety of the vaccine [38].

### 3.9. Nonreplicating Adenovirus Type-5 (Ad5) Vectored COVID-19 Vaccine

This vaccine was designed and developed based on the Admax system. A nonreplicating adenovirus (type-5) was used as a vector to carry the genetic information for expressing the SARS-CoV-2 spike proteins (Table 9). A cloning process was adopted to duplicate the genetic sequence of the ‘S' proteins, which was then incorporated into Ad-5 along with the tissue plasminogen activator signal peptide gene. Postvaccination analysis in the healthy volunteers indicated the presence of a high concentration of neutralizing antibodies such as CD4^+^ and CD8^+^. These antibodies were found to be expressed by TNF-*α* [39]. The levels of these antibodies were found to be dose-dependently varied and were very high upon comparison with the placebo group. The documented adverse events suggest that all the participants well tolerated the side effects without showing any major complications. However, the efficacy of the vaccine in older people (>55 years) was observed to be low with lower antibody responses after two dose vaccinations [40].

### 3.10. Gam-COVID-Vac Vaccine

The two recombination adenovirus vaccines are named rAD26-S and rAD5-S. The vaccines are designed and developed by a Russian pharmaceutical company called “Gameleya” (Table 10). A genetic sequence for the full-length glycoprotein ‘S' of the SARS-CoV-2 was recombined with adenovirus. The two vaccines were found to be more efficacious when they were mixed [41]. Volunteers receiving these vaccines showed no major adverse events, and their serological analysis revealed a 100% seroconversion rate and the presence of neutralizing antibodies on day 28. The analysis also indicated the presence of CD4+, CD8+, and IFN-*γ* in all the vaccine recipients. These antibodies demonstrate high efficacy against RBD of SARS-CoV-2 [42].

### 3.11. Ad26.Cov2.S Vaccine

Janssen Pharmaceutical has designed the vaccine based on the same principle that was used for the development of the Ebola vaccine (Table 11). The adenovirus vector Ad26 was used to carry the genetic sequence to the host cells. The company claims that the administration of a single dose of vaccine has produced neutralizing antibodies in 90% of vaccinated people after two weeks [43]. The vaccine in the clinical trials showed 66% of efficacy in protecting against the development of SARS-CoV-2 infection. The vaccine has also shown efficacy against the B, 1,351 variants of the virus [44]. Most of the study participants have shown no serious side effects and mild reactions are well tolerated. Pathological blood clotting is rarely seen in patients and is linked to low levels of platelets that trigger unexpected hypercoagulation [45]. One of the causes could be due to the wrong techniques of vaccine administration. If a vaccine enters the blood circulation in a large concentration, it may produce thrombocytopenia followed by hypercoagulability [46]. There has been no confirmed report of any of the COVID-19 vaccines having a negative impact on male and female reproductive systems, though some concerns have been expressed in the published literature [50].

## 4. Third (Booster) Dose of COVID-19 Vaccine

It is the additional dose of the COVID-19 vaccine after the protection (antibodies) levels start to wane. According to the available reports, the level of antibodies against COVID-19 started to decrease from 4–6 weeks postvaccination. The data is not clear about the role of the B-cell that normally stores the memory for synthesizing the antibodies against the antigens [52]. However, considering the severity of infection, a booster/third dose is recommended for all those patients who have weak immunity. FDA has suggested the third dose of Pfizer/Moderna for cancer, organ transplant, stem cell transplant, HIV, and other such patients who are under high dose of immunosuppressants. These patients were recommended to receive the booster dose after 28 days of the second dose [53]. Ideally, the same dose of the vaccine is recommended for the third dose, and more often it is done for those who took the mRNA vaccines. Studies in the past indicated that the administration of mRNA vaccines (Pfizer/Moderna) produced a weak immunological responses in patients suffering from immune system disorders [54].

Due to the appearance of mutant strains of SARS-CoV-2, clinical trials are also under progress to test the combined efficacy of COVID-19 vaccines. The pilot studies conducted after mixing the COVID-19 vaccines obtained from different sources have shown robust production of neutralizing antibodies in the test population [55]. One of the reasons reported is that the variation in vaccine technique might boost the immune system better without showing the tolerance towards the second dose of vaccine. However, there are reports indicating that such a combination may increase the complications. Important information about combinations of vaccines being trialed is represented in Table 12 [55–63].

Our observations from the review indicated that some of the COVID-19 vaccines have shown inflammatory reactions. The COVID-19 infection is associated with some risky inflammatory conditions such as vascular inflammation, myocarditis, and cardiac arrhythmias (Table 12). The binding of SARS-CoV-2 to ACE2 causes inflammation of the myocardium and lungs, causing injury to these organs [62]. One of the pathways for this is due to the release of several inflammatory mediators, partially because of ACE2 signaling. In previous studies, it was reported that the administration of vaccine for respiratory viruses such as influenza A and influenza B also produced inflammatory conditions. However, the effects of vaccination on the induction of inflammatory events in a few individuals need further research [63]. Furthermore, recent research has revealed that each vaccination has an almost similar degree of efficacy during clinical trials as well as when it is provided to the public (Table 13). Furthermore, multiple data (Table 14) shows that combining different vaccines during the second injection has no substantial detrimental impact.

## 5. Conclusion

COVID-19 vaccines have been safely administered to millions of people. All of the COVID-19 vaccines that have been approved have been thoroughly tested and are still being monitored. COVID-19 vaccines, like all vaccines, undergo a multistage testing process that includes large clinical trials involving thousands of individuals. These tests are intended to uncover any potential safety issues. This review examined the key data reported during the COVID-19 vaccine clinical trials. Despite the fact that the vaccines were developed using different technologies, they demonstrated a nearly identical ability to produce strong neutralizing antibodies against SARS-CoV-2 during clinical trials and in real-world practice among different segments of society. All of the vaccines were well tolerated, with only minor side effects. A few serious complications, including thrombocytopenia, anaphylaxis, myocarditis, and Guillain-Barre syndrome, were rarely observed in postvaccination people, but the exact cause was unknown. The duration of the immunogenic response, efficacy of the mutants' SARS-CoV-2 strains, and precise reasons for the life-threatening complications could not be confirmed based on the trial's data and need more in-depth investigation. Studies are also essential for determining the efficacy of vaccine combinations as well as the need for booster doses in the management of COVID-19. Further studies are required to determine these vaccines' efficacy against COVID-19 mutants like omicron.

## Figures and Tables

**Table 1:** Studies involving BNT162b2 mRNA COVID-19 vaccine (Pfizer-BioNTech COVID-19 vaccine).

Study name	Study type	Trial design	Efficacy	Safety and adverse drug reactions
A nucleoside-modified RNA encoding SARS-CoV-2 spike protein containing mutations which lock protein to confirmation, has shown by trials that it can induce both humoral and cellular immunity [19]				
Safety and efficacy of the BNT162b2 mRNA COVID-19 vaccine [19]	Phase 3, double-blind, randomized, controlled trial	Two doses of either 30 mcg of vaccine or placebo with 21 days apart were given and followed up for two months to 43,548 participants (≥16 years, healthy. randomized)	Among 36,523 participants who had no evidence of current or previous COVID-19, 8 in vaccine group and 162 in placebo group had confirmed COVID-19, 7 days after second dose vaccination, which gives vaccine efficacy of 95%	Among 8183 local site reactions (mainly, pain) with mild to moderate symptoms were higher in bnt162b2 group. Whereas systemic reaction was higher after second dose and in young vaccine recipients (aged 18–55 years old)
Age-dependent immune response to the BioNTech/Pfizer BNT162b2 COVID-19 vaccination [20]	Cohort study	A total of 176 people (60–80 years) were studied to compare their antibody responses to the first and second doses of the bnt162b2 mRNA COVID-19 vaccination	SARS-CoV-2 spike antibody titer after first (17 days) and second (7 days) dose was statistically higher in young group (<60 years old) whereas, after first dose only 4.4% (65.9% of elderly participants) of participants had titer below the cut point with no participant tested below the cut point after the second dose	After first vaccination, 51.6% of younger and 93.9% in elderly participants reported no symptoms while the remaining reported mild symptoms. After second dose, 35.3% of young participants reported symptoms up to score 6 and only 17% of elderly participants reported symptoms up to 3
B and T cell immune responses elicited by the BNT162b2 (Pfizer–BioNTech) 2 COVID-19 vaccine in nursing home residents [21]		Sixty nursing home residents (NHR) (median age 87.5) were recruited, 18 of whom had never been infected with SARS-CoV-2. SARS-CoV-2-S targeting antibody and functional T-cell responses were the major outcomes.	In convalescent NHR, plasma antibody levels and SARS-CoV-2 S-reactive IFN-*γ* CD8+ and/or CD^4+^ 41 T cells were greater. The percentage of NHR with detectable SARS-CoV-2 IFN-*γ* CD8+ or CD4+ T-cell responses (or both) declined consistently after vaccination.	In NHR, the BNT162b2 COVID-19 vaccination induces strong SARS-CoV-2-S antibody responses
Safety, immunogenicity, and efficacy of the BNT162b2 COVID-19 vaccine in adolescents [22]	Multinational, placebo-controlled, observer-blinded trial	2260 adolescents aged 12–15 years old were randomly assigned (1 : 1) to receive two doses of either BNT162b2 or placebo with 21 days apart	Seven days after second dose of Pfizer vaccine showed efficacy of 100%	Only mild-moderate adverse events at injection-site pain, fatigue, headache with no severe vaccine related adverse events
BNT162b2 mRNA COVID-19 vaccine in a nationwide mass vaccination setting [23]	Observational study	A total of 596,618 participants (≥16 years old) who were vaccinated from December 2020 till February 2021 were matched to nonvaccinated participants (1 : 1 ratio) with a total of 1,163,534 participants enrolled in study	The vaccine has a 60% efficacy against SARS-CoV-2, 70% against severe COVID-19 illness, and 84% against COVID-19 death 21–27 days after the first dose. The vaccine efficacy, 7 days after second, against COVID-19 infection, hospitalization, severe disease, and death was 92%, 94%, 87% and 92%, respectively.	The BNT162b2 mRNA vaccine protects against a variety of Covid-19-related outcomes

**Table 2:** Studies involving CoronaVac COVID-19 vaccine.

Study name	Study type	Trial design	Efficacy	Safety and adverse drug reactions
CoronaVac is an inactivated vaccine candidate against SARS-CoV-2 and has shown effective immunogenicity in animals and human by inducing both humoral and cellular immunity [7, 8]				
Interim report: safety and immunogenicity of an inactivated vaccine 1 against SARS-CoV-2 in healthy Chilean adults in a phase 3 clinical trial [24]	Phase III single-blind randomized control trial	434 participants were randomly assigned to either receive CoronaVac vaccine (270) or placebo (164). The main aim of the study was to determine adverse events that occur 7 days after each dose.	At day 14 postimmunization, the seroconversion rate for RBD-S igg in young patients (18–59 years old) was 47.8%, whereas in elderly (>60 years old) 18.1%. On day 42, it was 95.6% in young and 87.5% in elderly participants.	Most local common adverse effect was injection site pain, which was statically higher among vaccine group compared to placebo A total of 55 unsolicited adverse effects including gastrointestinal discomfort, abdominal pain, odynophagia, and back pain were reported
Safety, tolerability, and immunogenicity of an inactivated SARS-CoV-2 vaccine (CoronaVac) in healthy adults aged 60 years and older: a randomized, double-blind, placebo-controlled, phase 1/2 clinical trial [25]	Randomized, double-blind, placebo-controlled, phase 1/2 clinical trial	Phase 1 trial 72 participants (≥60 years old) were randomized (2 : 1) to receive either CoronaVac (3 or 6 mcg) or placebo. 350 people were randomized (2 : 2:2 : 1) to receive either 15 mcg, 3 mcg, or 6 mcg per dose of vaccination, or placebo, in phase 2 trials.	In phase 1 trial, seroconversion rate in two doses of 3 mcg of CoronaVac was 100% while 95.6% in 6 mcg CoronaVac arm without any statistical difference between them In phase 2 trial, seroconversion rate did not show statistical difference between 3 mcg and 6 mcg	Out of 422 participants in both trials, 87% of them had at least one adverse event. Pain at the injection was main adverse event. In phase 2 trial. 8 serious adverse reactions were reported (all in vaccine arms and none in placebo) However, none of them were considered as vaccine related
Effectiveness of CoronaVac in the setting of high SARS-CoV-2 P.1 variant transmission in Brazil: a test-negative case-control study (Preprint) [26]	Case-control study	53,176 healthy healthcare workers (hcws, ≥18 years old) were enrolled, 46,884 (88%) received at least one dose of CoronaVac vaccine	CoronaVac immunization with at least one dose was linked with a 50% reduction in symptomatic COVID-19 infection after 14 days or more. While it did not show reduction in risk for COVID-19 infection	Not documented
Immunogenicity and safety of a SARS-CoV-2 inactivated vaccine in healthy adults aged 18–59 years: Report of the randomized, double-blind, and placebo-controlled phase 2 clinical trial [preprint] [27]	Randomized, double-blind, and placebo-controlled phase 2 clinical trial	On a day 0,14 or day 0,28 schedule, 600 healthy adults (18–59 years old) were randomly randomized (2 : 2 : 1) to receive 2 doses of 3 mcg or 6 mcg of CoronaVac or placebo	Both schedules had 90% increase in seroconversion rate with no significance difference between them. A 3 mcg vaccine showed a 92.4% in 0,14 schedule and 97.4% in 0, 28 schedule.	On day 0, 14 schedule vaccine administration 6 mcg showed higher incidence of adverse effects compared to 3 mcg of vaccine. While on *n* day 0, and day 28 schedules, 3 mcg, 6 mcg, and placebo had no statistical difference in adverse effects. All adverse reactions were mild to moderate intensity.
Safety, tolerability, and immunogenicity of an inactivated SARS-CoV-2 vaccine in healthy adults aged 18–59 years: a randomized, double-blind, placebo-controlled, phase 1/2 clinical trial [28]	Randomized, double-blind, placebo-controlled, phase 1/2 clinical trial	In phase 1, 144 participants (≥18 years old) were randomly assigned to 2 cohort day 0, 14 and 0, 28 schedules; within each cohort people were assigned for block 1 (3 mcg of vaccine or placebo) or block 2 (received either 6 mcg of vaccine or placebo). While in phase 2 study 600 participants were randomly assigned in two cohorts (0, 14 and 0, 28) and randomly assigned (2 : 2 : 1) to receive either 3 mcg, 6 mcg, or placebo	In phase 1 trial, the seroconversion rate of three arms (3 mcg, 6 mcg, and placebo) was 46%, 50%, 0%, respectively, in days 0, 14 schedule, while it was higher in days 0, 28 schedule of 83%, 79%, 4%, respectively In phase 2 trials, it was seen for 92%, 98%, 3% respectively in 3 mcg, 6 mcg, and placebo arms in days, 0,14 schedule, whereas 0, 28 days schedule had a seroconversion rate of 97%, 100%, 0% among the three arms respectively	In phase 1 trial, the adverse reactions occurrence was higher in 6 mcg vaccine arm compared to 3 mcg, and placebo in the days 0, 14 cohort, while 0, 28 cohort reported lower incidence of adverse reaction in the vaccine arms 3 mcg, 6 mcg vaccine, or placebo of 13%, 17%, 13%, respectively In phase 2 study, the adverse reaction occurrence in 3 mcg, 6 mcg, and placebo was 33%, 35%, 22%, respectively in days 0,14 cohort. While days 0, 28 cohort reported lower incidence of adverse reactions of 19%, 19%, 18%, respectively

**Table 3:** Studies involving ChAdOx1 nCoV-19 vaccine.

Study name	Study type	Trial design	Efficacy	Safety and adverse drug reactions
ChAdOx1 nCoV-19 is a replication-deficient chimpanzee adenoviral vector Containing the SARS-CoV-2 structural surface glycoprotein antigen gene, has shown induction of humoral and cellular immunity [28]				
Safety and efficacy of the ChadOx1 nCoV-19 vaccine (AZD1222) against SARS-CoV-2: an interim analysis of four randomized controlled trials in Brazil, South Africa, and the UK [29]	Interim analysis of four randomized controlled trials	This interim analysis included data from four ongoing randomized control trails (three single blinded and one double blinded). 23,848 participants (≥18 years old) received vaccination and 11636 of them received two doses of either standard dose of ChAdOx1 nCoV-19 or placebo (5807 vs 5829, respectively); in UK subset, they received half dose in first shot and standard dose in their second shot). The primary objective was to determine the efficacy of ChAdOx1 nCoV-19 against COVID-19	11636 were included in the analysis, total vaccine efficacy was 70.4% (62.1% among those who received two standard doses whereas 90% among participants who received half dose during first shot vaccine. 3 weeks after vaccination, 11 cases in placebo arm were hospitalized from COVID-19 (2 considered as severe COVID-19) and none in vaccine group.	A total of 175 adverse events were reported; 3 of them were considered related to the intervention (vaccine or placebo); one case was in vaccine arm, one in placebo arm, and one case who remained masked to group allocation
Thrombosis and thrombocytopenia after ChAdOx1 nCoV-19 vaccination [30]	Case report	A case report of 5 healthcare workers who received ChAdOx1 nCoV-19 vaccination	—	7–10 days after receiving first dose of CHADOX1, high levels of antibodies to platelet factor 4-polyanion complexes were documented in all patients without any previous exposure to heparin

**Table 4:** Studies involving mRNA-1273 (Moderna vaccine).

Study name	Study type	Trial outcome and design	Efficacy	Safety and adverse drug reactions
MRNA-1273 vaccine an encapsulated lipid-nanoparticle (LNP) mRNA expressing spike protein has shown efficacy in animals and encouraging safety and efficacy profile in human subjects [30]				
Efficacy and safety of the mRNA-1273 SARS-CoV-2 vaccine [31]	Phase 3 randomized, observer-blinded, placebo-controlled trial	A total of 30,420 participants (aged 18) were randomized to receive two doses of mRNA-1273 (100 mcg) or placebo, 28 days apart. COVID-19 prevention was the major goal	Out of 30,420 participants, 96% of them received two injections and 2.2% had positive COVID-19 at baseline. Out of all participants, 11 cases in the vaccine arm and 185 were diagnosed with COVID-19 infection indicating 95% of vaccine efficacy against symptomatic COVID-19 infection	In comparison to placebo, the vaccine group reported more solicited injection site reactions after the first dose and the second dose and in younger adults than older adults

**Table 5:** Studies involving BBIBP-Corv vaccine (Sinopharm COVID-19 vaccine).

Study name	Study type	Trial design	Efficacy	Safety and adverse drug reactions
BBIBP-Corv is an inactivated SARS-CoV-2 virus (HB02 strain) that has showed effectiveness in inducing both humoral and cellular immunity [31]				
Safety and immunogenicity of an inactivated SARS-CoV-2 vaccine, BBIBP-Corv: a randomized, double-blind, placebo-controlled, phase 1/2 trial [32]	Randomized, double-blind, placebo-controlled, phase 1/2 trial	Both 18-59- and 60-years old cohorts received either vaccine (2 mcg, 4 mcg, or 8 mcg) or placebo in the phase 1 trial. 18–59 years old were randomized and recruited in a phase 2 trial to receive either a placebo or a single dose (8mcg) or double dosage (8 mcg) of the vaccine (4 mcg on day 0 and 14 or 21 or 28)	The younger cohort (18–59 years old) reached an earlier 100% seroconversion rate than older group (≥60 years old). 4 and 8 mcg vaccine group reached a 100% seroconversion rate on day 28 while the 2-mcg group reach it on day 42.	Young participants (8%) had more adverse events than older participants (4%) and young participants who got a lower vaccine dosage had more adverse events than older participants (4%)

**Table 6:** Studies involving BBV152 vaccine.

Study name	Study type	Trial design	Efficacy	Safety and adverse drug reactions
Bbv152 is a whole-virion *β*-propiolactone-inactivated SARS-CoV-2 vaccine (niv-2020-770 strain) and formulated with Algel-IMDG adjuvant. Based on preclinical trial bbv152 showed an enhancement in both humoral and cell-mediated immune response [33]				
Safety and immunogenicity of an inactivated SARS-CoV-2 vaccine, BBV152: interim results from a double-blind, randomized, multicenter, phase 2 trial, and 3-month follow-up of a double-blind, randomized phase 1 trial [34]	Phase 1&2 randomized multicenter double-blind trials	380 participants (12–65 years old) were randomly assigned (1 : 1) to receive either 3 mcg or 6 mcg of vaccine at day 0 and 28	The seroconversion among the 6 mcg and 3 mcg vaccine groups was reported in 98.3% and 92.9% of, respectively. While the seroconversion in 96·6% among 6 mcg group	Injection site pain was the most common among socialized adverse reactions

**Table 7:** Studies involving RBD-based protein subunit vaccine.

Study name	Study type	Trial design	Efficacy	Safety and adverse drug reactions
Zf2001 is a protein subunit vaccine targets the receptor binding domain (RBD) of the SARS-CoV-2s protein produced in Chinese hamster ovary (CHO) cells adjuvanted with aluminium hydroxide [35]				
Safety and immunogenicity of a recombinant tandem-repeat dimeric RBD-based protein subunit vaccine (ZF2001) against COVID-19 in adults: Two randomized, double-blind, placebo-controlled, phase 1 and 2 trials [36]	Phase 1 and phase 2 randomized, double-blind, placebo-controlled trials	50 participants (18–59) years old were randomly assigned (2 : 2 : 1) to receive either placebo, 25 mcg vaccine, or 50 mcg vaccines in the phase 1 trial In the phase 2 trail, 900 participants with three groups received two vaccine doses (25 mcg or 50 mcg) or placebo, and three groups receiving three vaccine doses (25 mcg or 50 mcg) or placebo	Seroconversion rate was among participants who received three doses of placebo, 25 mcg vaccine, 50 mcg vaccine (0%, 97%, 93%) were higher than those who received only two doses (1%, 83%, 73)	In both trials, the majority of participants had mild to moderate adverse effects A total of 7 participants had severe adverse effects but none of them were vaccine related

**Table 8:** Studies involving EpiVacCorona vaccine.

Study name	Study type	Trial and design	Efficacy	Safety and adverse drug reactions
A chemically synthesized immunogens corresponding to EpiVacCorona is a chemically synthesized peptide immunogens of protein S in conjugation with recombinant SARS-CoV-2 protein S, which showed high immunogenicity in preclinical studies [37]				
A single blind, placebo-controlled randomized study of the safety, reactogenicity and immunogenicity of the “EpiVacCorona” vaccine for the prevention of COVID-19, in volunteers aged 18–60 years (phase I–II) [38]	Phase I-II single blind randomized clinical trial	Phase 1 trial enrolled 14 participants aged 18–30 years while in phase 2 trial a total of 86 participants were randomly enrolled to receive 2 doses of either vaccine or placebo spaced 21 days apart	On day 42 post first dose, vaccinated participants reached a 100% seroconversion rate for the vaccine antigen and 82.1% IGg seroconversion rate, while none of the placebo group had seroconversion	Both in phase 1 and phase 2 trials, injection site pain was observed in the small number of participants Only one case had a moderate fever and headache 12 hr. after vaccination

**Table 9:** Studies involving nonreplicating adenovirus type-5 (Ad5) vectored COVID-19 vaccine.

Study name	Study type		Trial design	Efficacy	Safety and adverse drug reactions
A vectored defective replicating adenovirus type-5 expressing the spike glycoprotein SARS-CoV-2 virus, has been shown acceptable safety and tolerability profile and promising immunogenicity results in phase 1 trial [39]					
Immunogenicity and safety of a recombinant adenovirus type-5-vectored COVID-19 vaccine in healthy adults aged 18 years or older: a randomized, double-blind, placebo controlled, phase 2 trial [40]	Phase 2 double blind randomized controlled trial	A total of 508 healthy participants (>18 years old) were randomly assigned (2 : 1 : 1) to receive the vaccine (1 × 10^11^ viral particles, 5 × 10^10^ viral particles) or placebo, respectively		On day 28, seroconversion rate was shown in 96% of the 1 × 10^11^ viral particles group and 97% of the 5 × 10^10^ viral particles group. While the seroconversion to live SARS-CoV-2 virus was detected in 59% of the 1 × 10^11^ viral particles group and 47% of the 5 × 10^10^ viral particles group	Fatigue, headache, and fever were the most often reported side effects. While the pain was the most common local adverse response

**Table 10:** Studies involving gam-COVID-Vac vaccine.

Study name	Study type	Trial design	Efficacy	Safety and adverse drug reactions
Gam-COVID-Vac is a combined vector vaccine carrying full gene for SARS-CoV-2 glycoprotein S based on rAd type 26 (rAd26) and rAd type 5 (rAd5). Phase 1/2 trial showed a well-tolerated and high immunogenicity of the vaccine in healthy adults [41]				
Safety and efficacy of a rAd26 and rAd5 vector-based heterologous prime-boost COVID-19 vaccine: an interim analysis of a randomized controlled phase 3 trial in Russia [42]	Phase 3 randomized controlled trial	21977 participants (>18 years old) were randomly assigned (3 : 1) to receive either vaccine (*n* = 16501) or placebo (*n* = 5476); 19866 of them received two doses of either vaccine or placebo with 21 days apart	21 days after the first vaccination, Gam-COVID-Vac showed an efficacy of 91%. Interestingly, vaccine efficacy was 91.8% in elderly participants while it was more than 78% in all ages.	Headache, injection-site reaction, and asthenia were the most common recorded symptoms. None of the serious adverse events were related to COVID-19 vaccine.

**Table 11:** Studies involving Ad26.COV2.S (Johnson & Johnson COVID-19 vaccine).

Study name	Study type	Trial design	Efficacy	Safety and adverse drug reactions
Ad26.COV2.S is a viral vector vaccine based on adenovirus type 26 encoding a full length of SARS-CoV-2 spike protein. It showed in preclinical and phase 1 trials a good safety and immunogenicity profile [43, 44]				
Safety and efficacy of single-dose Ad26.COV2.S vaccine against COVID-19 [45]	Double-blinded randomized control trial	Ad26.COV2.S (5 × 10^10^ viral particles) or placebo were given to 19,630 SARS-CoV-2-negative individuals (18 years old) who were randomly randomized (1 : 1) to receive a single dose of Ad26.COV2.S (5 × 10^10^ viral particles) or placebo	Ad26.COV2.S had a 66.9% efficacy at onset of 14 days and 66.1% efficacy at onset of ≥28 days, but it had a higher efficacy against severe COVID-19 infection with a 76.7% efficacy	Seven severe adverse reactions were classified as vaccine related. Three deaths happened in the vaccinated group and 16 in the placebo group (none of them were considered related to vaccine or placebo)
Thromboembolic events in the South African Ad26.COV2.S vaccine study [46]	An open label, single-group, phase 3b implementation study	A total of 288,368 healthcare workers (>18 years old) received one dose of Ad26.COV2.S vaccine was enrolled	—	81% of reported adverse events were mild to moderate intensity while 50 of them had adverse events classified as a severe or special interest

**Table 12:** Summary of common side effects and rare side effects associated with COVID-19 vaccines.

Name of vaccine	Common side effects	Rare side effects
BioNTech vaccine [20]	Fever, muscle pain, chills, fatigue	Myocarditis, appendicitis, angioedema
CoronaVac [24]	Headache, fatigue, diarrhea, pain at injection site	Ocular congestion, muscle spasm, hyposmia, nosebleed
ChadOx1 [29]	Chills, fever, joint pain, fatigue, headache	Thrombocytopenia, anaphylaxis
mRNA 1273 [31]	Stiffness of muscle, chills, lymphadenopathy, pain	Inflammation of pericardium, hypersensitivity
BBIBP CorV [32]	Flushing, swelling, fever, headache	Nasopharyngitis, drowsiness, palpitation
BBV152 [34]	Headache, fatigue, fever	Hypersensitivity, dizziness, difficulty in breathing
RBD (ZF2001) [36]	Cough, itching, headache, fever	Rhabdomyolysis, impaired appetite, hypersensitivity
EpiVac [38]	Sore arm, tiredness, fever, headache	Not documented
Ad-5 (Admax) [40]	Fatigue, headache, fever	Not documented
Gam-COVID-Vac [42]	Weakness, myalgia, headache, pain at the site of injection	Deep vein thrombosis, hemorrhagic stroke, hypertension
Johnson and Johnson [46]	Headache, chills, fever, muscle pain	Thrombocytopenia syndrome, Guillain–Barre syndrome (an autoimmune disorder of nervous system)

**Table 13:** Comparison of efficacy and effectiveness of important COVID-19 vaccines [51].

Name of vaccine	Efficacy (clinical trials) (%)	Effectiveness (real-world)
BioNTech vaccine	94	87.9%
CoronaVac	95	88.7%
ChadOx1	74	88%
mRNA 1273	78	__
BBIBP CorV	62	49.6%
Johnson and Johnson	66	76.7%

**Table 14:** Studies involving combination vaccines.

Study name	Study type	Trial design	Efficacy	Safety and adverse drug reactions
BNT162b2 (Pfizer–BioNTech) vaccine and the mRNA-1273 (Moderna) vaccine				
Preliminary findings of mRNA COVID-19 vaccine safety in pregnant persons [56]	Observational study	A total of 35,691 pregnant women (≥16 years old) who received either Pfizer or Moderna vaccines were included using “v-safe after vaccination health checker” system	—	Overall, reactogenicity between pregnant and nonpregnant women were similar except for injection-site pain, which was reported more in pregnant. The most reported adverse events are headache, myalgia, chills, and fever. Among 221 pregnancy-related reported adverse events, the most common one was spontaneous abortion (46 cases)
COVID-19 vaccine response in pregnant and lactating women: a cohort study [57]	Cohort study	Participants in the U131 reproductive-age vaccine study were given either the Pfizer or the Modern vaccine. In comparison to nonpregnant women, the primary goal was to assess the immunogenicity and reactogenicity of the mRNA vaccination in pregnant and lactating women	Vaccine induced antibodies titers were higher among pregnant and lactating compared to nonpregnant adults which were detected all in breastmilk and umbilical cord. The second vaccine dose showed a higher IgG titer but not IgA in maternal blood and breastmilk	—
The vaccine-elicited immunoglobulin profile in milk after COVID-19 mRNA-based vaccination is IgG-dominant and lacks secretory antibodies [58]	Cohort study	A total of 10 participants who received either Pfizer or Moderna vaccines were enrolled. The main aim was to assess the presence of specific antibodies (IgG, IgA) in milk against the SARS-CoV-2 virus before and after the mRNA vaccine.	Upon results, postvaccine (day 14) IgA antibody was positive in 60% of participants, and 100% of them had significant levels of IgG antibody in breastmilk. Furthermore, a spike-specific secretory antibody was shown in 50% of participants' breastmilk	—
BNT162b2 (Pfizer–BioNTech) and ChAdOx1 nCoV-19 (Oxford–AstraZeneca; ChAdOx1)				
First-dose ChAdOx1 and BNT162b2 COVID-19 vaccines and thrombocytopenic, thromboembolic and hemorrhagic events in Scotland [59]	Observational study	2.53 million participants (≥18 years old) received either ChAdOx1 (1.71 million) or BNT162b2 (0.82 million)	—	27 days post-ChAdOx1 vaccine showed an increased risk of arterial thromboembolic events. On the other hand, the BNT162b2 vaccine did not show any association with thromboembolic events
Impact of vaccination on new SARS-CoV-2 infections in the United Kingdom [60]	Cohort study	383,812 participants (≥18 years old) who were received either ChAdOx1 or BNT162b2 vaccines were enrolled	21 days after vaccination ChAdOx1 and BNT162b2 decreased the incidence of the new SARS-CoV-2 infection by 61% versus 66%, respectively. While after second dose, they showed a higher protection up to 79% versus 80%, respectively.	—
BBV152 vaccine (COVAXIN) and ChAdOx1 nCoV-19 (Oxford–AstraZeneca; ChAdOx1)				
Antibody response after first dose of ChAdOx1 nCoV-19 (Covishieldtm®) and BBV-152 (COVAXINtm®) amongst healthcare workers in India: preliminary results of cross-sectional coronavirus vaccine-induced antibody titre (COVAT) study [61]	Cross-sectional study	552 healthcare workers (≥18 years old) with or without a history of SARS-CoV-2 were included in the study and received their first dose of covishield (456) or COVAXIN vaccine (452) (96)	Out of 552 participants who received either covishield or COVAXIN, 79% of them were seropositive and responders for antispike antibodies. However, the covishield vaccine showed a significantly higher rate of respondence compared to COVAXIN.	Among 552 participants, Covishield vaccine showed a significantly higher incidence of adverse events compared to COVAXIN

## Data Availability

The data used to support the findings of this study are included within the article.
